# Super flame-retardant lightweight rime-like carbon-phenolic nanofoam

**DOI:** 10.1038/srep33480

**Published:** 2016-09-15

**Authors:** Haiming Cheng, Changqing Hong, Xinghong Zhang, Huafei Xue, Songhe Meng, Jiecai Han

**Affiliations:** 1 National Key Laboratory of Science and Technology on Advanced Composites in Special Environments, Harbin Institute of Technology, Harbin 150001, P.R. China

## Abstract

The desire for lightweight nanoporous materials with high-performance thermal insulation and efficient anti-ablation resistance for energy conservation and thermal protection/insulation has greatly motivated research and development recently. The main challenge to synthesize such lightweight materials is how to balance the relationship of low thermal conductivity and flame retardancy. Herein, we propose a new concept of lightweight “rime-like” structured carbon-phenolic nanocomposites to solve this problem, where the 3D chopped network-structured carbon fiber (NCF) monoliths are incorporated with nanoporous phenolic aerogel to retain structural and functional integrity. The nanometer-scaled porous phenolic (NP) was synthesized through polymerization-induced phase separation and ambient pressure drying using phenolic resin (PR) solution as reaction source, ethylene glycol (EG) as solvent and hexamethylenetetramine (HMTA) as catalyst. We demonstrate that the as-prepared NCF-NP nanocomposite exhibits with a low density of 0.25–0.35 g/cm^3^, low thermal conductivity of 0.125 Wm^−1^K^−1^ and outstanding flame retardancy exceeding 2000 °C under arc-jet wind tunnel simulation environment. Our results show that the synthesis strategy is a promising approach for producing nanocomposites with excellent high-temperature heat blocking property.

Traditional carbon fiber reinforced phenolic resin (C-Ph) composites have played an important and strategic role in the field of energy efficiency for buildings and thermal protection/insulation^1^,^2^,^3^,^4^,^5^. However, C-Ph composites have relatively high density and thermal conductivity, owing to its densified structure consisting of dispersive carbon fiber and thermosetting phenolic resin. Lightweight nanostructured organic-inorganic hybrid composites have received increasing attentions applied in the energy conversation, fire-resistant construction and chemical engineering^6^,^7^,^8^,^9^,^10^, because of the possibility to combine the enormous functional variation of organic chemistry with the advantages of a thermally stable and robust inorganic substrate^11^. The symbiosis of organic and inorganic components can lead to outstanding material properties, which differing considerably from their individual or isolated components^12^,^13^.

Quasi-stable, low-density, three-dimensional assemblies of nanoparticles are referred to as aerogels and typically are derived from drying wet-gels by supercritical, subcritical or ambient pressure to remove the pore-filling solvent and retain its formed skeleton structure simultaneously^14^,^15^. Their large internal nanoporous void space is responsible for ultra-low thermal conductivity and thermal insulation application. Recently nanostructured organic polymer materials, particularly those constructed with uniform nanopores in inorganic matrix, have received interesting attentions in materials science^16^,^17^,^18^. However, pure organic aerogels constructed by conventional approaches are easily brittle in the aspect of mechanical strength and stiffness preserving.

Inspired by “rime-like” structure in nature, we describe lightweight (0.25–0.35 g/cm^3^), highly porous NCF-NP composites that are produced by impregnating nanostructured NP sol into 3D porous NCF framework by polymerization-induced phase separation of PR, EG and HMTA (Fig. 1a), and subsequent ambient pressure drying. The anisotropic NCF-NP nanofoam is mechanical stiff and without presence of shrinkage after dying. It has been found that NP easily occupies the space void of NCF and also adequately coats the surface of carbon fiber (Fig. 1b). The resultant nanocomposites exhibit sufficiently low thermal conductivity and also show high flame retardancey.

## Results

### Concept of synthesizing route for rime-like NCF-NP nanofoam

**Figure 1:**
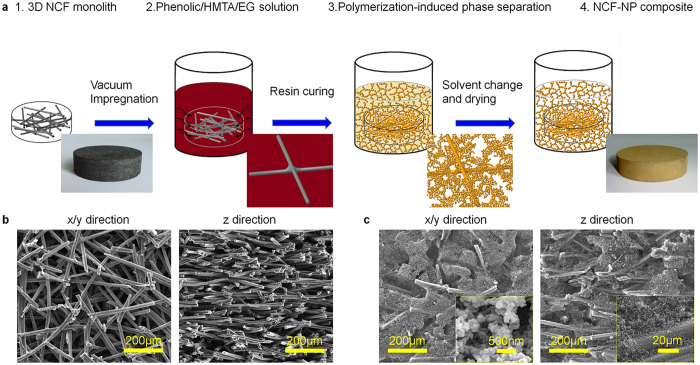
Schematic preparation diagram of NCF-NP nanocomposite foam. (**a**) Specific synthesized pathway and steps: 1. NCF with chopped carbon fiber network. 2. Vacuum impregnation of NCF composite in PR/HMTA/EG solution. 3. Gelation and polymerization in PR/HMTA/EG solution. 4. “Rime-like” NCF-NP nanocomposite after curing, IPA exchange and drying under ambient pressure. (**b**) Representative SEM image of a NCF framework in *xy* and *z* direction. (**c**) SEM image of NCF-NP nanocomposite in *xy* and *z* direction. Inset: higher magnification image of phenolic nanofoam in the space void between fibers and on the surface of fibers.

Figure 1 describes the synthesis pathway. A fibrous 3D NCF monolith (step 1, Fig. 1a) with a low density (~0.192 g/cm^3^) and high porosity (~88.0%) is adopted owing to its low thermal conductivity and controllable microstructure, which is prepared by pressure filtration technique^19^. The chopped carbon fibers in NCF framework distribute with network structure in xy direction and quasi-layered distribution in z direction (Fig. 1b). The 3D NCF is first immersed and impregnated in phenolic resin/HMTA/EG solution (step 2, Fig. 1a). The impregnated NCF substrate is then heated at 90 °C for 1 h, and then 120 °C for 3 h, and lastly 180 °C for 3 h to make the mixture solution gel and cure (step 3, Fig. 1a). The cured NCF-NP composite was conducted to immerse in IPA to exchange redundant EG and directly dried under ambient pressure (step 4, Fig. 1a), and the final NCF-NP composite had a relatively low density of 0.25–0.35 g/cm^3^. Homogeneous phenolic nanofoam in NCF framework without macroscopic cracks and agglomeration was achieved. Micrographs of the as-prepared nanocomposites show that the phenolic nanofoam uniformly occupies the void volume between fibers, and adequately coats the surface the fibers (Fig. 1c). The constructed NCF-NP nanocomposite exhibits a rime-like morphology, in which the NP nanofoam has physically wrapped via van der Waals forces and partially chemically bonded on 3D NCF network with interconnected pore size of 20~40 nm^20^.

### Control of pore structure for NP

**Figure 2:**
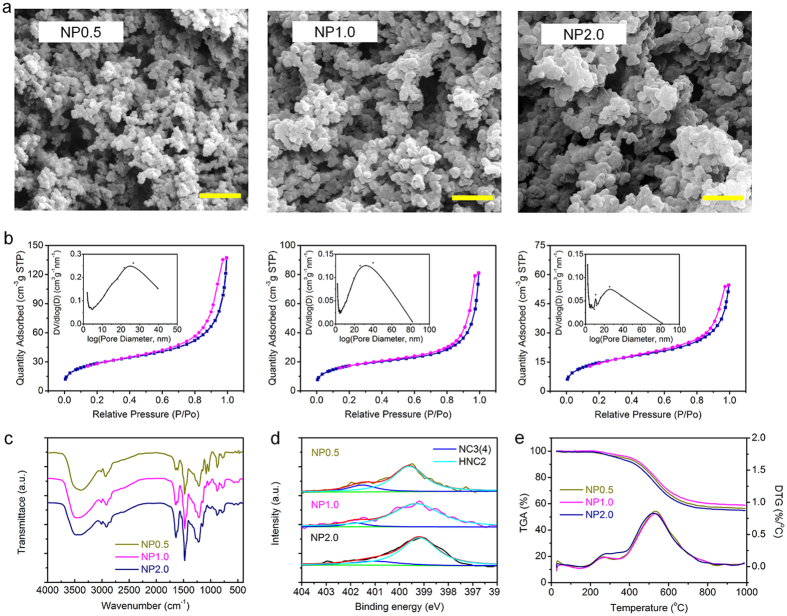
Porous NP synthesized with different HMTA content. (**a**) SEM micrographs: sample NP0.5, NP1.0 and NP2.0. Scale bars, 500 nm. (**b**) N_2_ adsorption–desorption isotherms at 77 K, STP = standard temperature and pressure, P/P_o_ = the ratio of the adsorption pressure (P) and the saturation vapor pressure (P_o_). Insets show quite broad BJH desorption plots, they look like overlapping pore-size distributions. (**c**) FTIR spectra reveal HMTA functioned not only as a catalyst but also as a cross-linking reagent into the cured resin. (**d**) TGA and DTG curves from room temperature to 1000 °C with a heating rate of 10 °C/min under argon atmosphere. (**e**) N1s XPS from the porous phenolic resin.

The as-prepared NP nanofoams have the typical bi-continuous and percolating structures that prepared by polymerization-induced phase separation^21^, in which the formed rime-like phenolic aggregates attached to the fibers upon gelation, and the nanopores were maintained after NP curing^22^. The pore structures for NP nanofoam can be controlled by changing the content of HMTA from 0.5% (sample NP0.5) to 1.0% (sample NP1.0) and 2.0% (sample NP2.0) while keeping the EG weight fraction constant at 80% (Supplementary Table S1). The examined value of S_BET_, S_mic_ and S_meso_ decreases with the increasing of HMTA content (Table 1), which is in agreement with the variation of pore volume for the three samples (inserts in Fig. 2). Furthermore, the particle size of NP increases with increasing of HMTA addition (see Fig. 2a), and the NP aerogel grains became more aggregated and coarse, leading to the appearance of more macropores and thus less micro- and mesopores simultaneously. However, the interior of NP nanofoam still retrains large mesopores (S_meso_ from 90.55 to 40.36 m^2^/g) and small micropores (S_meso_ between 5.356 and 0.614 m^2^/g). Neither the surface nor the interior pore structure resembles typical aerogels in which the pore structure is predominantly mesoporous. The N_2_ adsorption-desorption isotherms (Fig. 2b) show type IV nitrogen isotherms with a H3 hysteresis loop at high relative pressure according to the IUPAC classification^23^,^24^,^25^. The pore size distribution by the BJH method show that the pore size mainly distributes between 20 nm and 40 nm with an increasing concentration of HMTA from 0.5% to 2.0%.

The FTIR spectra analysis is depicted in Fig. 2c. The adsorption peaks at 2924, 2867 and 1477 cm^−1^ are associated with -CH_2_- stretching vibrations^26^,^27^,^28^, while the peaks at 1455, 1277, 1204, 1148, 1083 and 1038 cm^−1^ are attributed to -CH_2_-O-CH_2_- stretching vibrations of methylene ether bridges between benzene rings^29^,^30^. Simultaneously, the aromatic C-H out-of-plane deformation vibrations (823, 758 and 730 cm^−1^, respectively) become gradually smaller in transmittance with the increased HMTA content, indicating more aromatic nuclei are cross-linked by the methylene (-CH_2_-) and dibenzyl ether (-CH_2_-O-CH_2_-) bridges in the NP nanofoam. The absorption peaks at 3467, 1630, 1540, 1247 and 762 cm^−1^ corresponded to stretching vibration of N-H (3467 and 762 cm^−1^), the deformation (1630 cm^−1^) and stretching vibration (1247 cm^−1^) of C-N^31^,^32^,^33^,^34^. It is noticeable that the content of the N-containing functional groups increases with the amount of HMTA, and the characteristic absorption peak (945 cm^−1^) of benzoxazine does not appear, revealing that N-methylene from HMTA was introduced into the backbone of the phenolic nanofoam^35^,^36^,^37^.

The N1s XPS spectra analysis were found the appearance of two typical peaks: the HNC2 group at 399.6 eV and the NC3(4) group at 401.0 eV^38^,^39^,^40^. The group of HNC2 and NC3(4) are the characteristic structure of N-methylene bridges and tertiary/quaternary nitrogen, respectively, which is the main intermediate of the cured NP nanofoam^41^,^42^. The HNC2 is the major product and the mole fraction reaches up to 81% for NP0.5, and even over 92% for NP1.0, accounting for the higher T_5%_, T_10%_, T_d.max_ and char yield (Table 1). The carbon, oxygen and nitrogen peaks were observed in the full-scan XPS spectra of sample NP0.5, NP1.0 and NP2.0 (Supplementary Fig. S1), and the nitrogen content increases proportionally with the HMTA content (Supplementary Table S2 and S3). The initial decomposition temperature T_5%_ and T_10%_ for the sample NP1.0 are higher than that of NP0.5 and NP2.0, and the T_d,max_ reaches 536.3 °C, which are increased by 6.1 and 15.9 °C, respectively (Fig. 1f and Table 1). Furthermore, the maximum char yield of 58.9% was obtained for NP1.0, showing that the amount of HMTA has an important effect on the char yield and ablation-resistance of the cured NCF-NP nanofoam^43^ (Supplementary Fig. S2 and Table S6).

### Materials properties for NCF-NP composites

**Figure 3:**
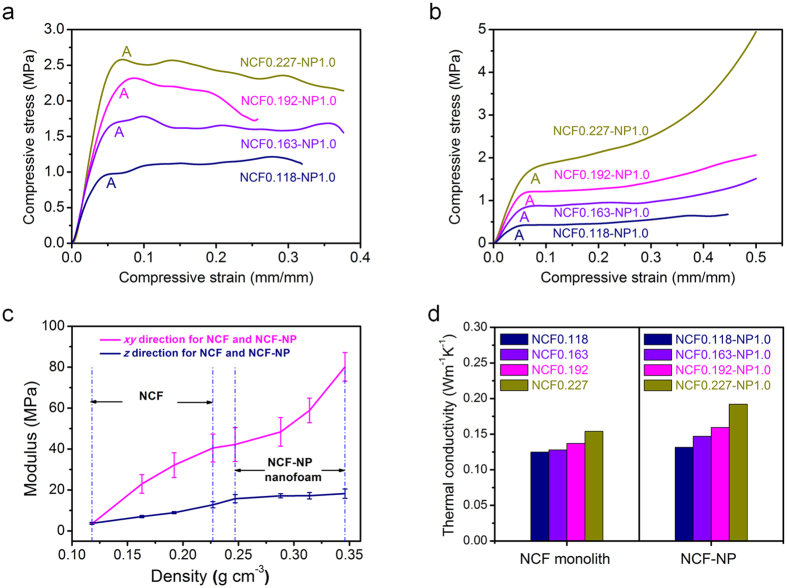
Mechanical properties and thermophysical parameters of NCF and NCF-NP nanofoams. (**a,b**) Representative compressive stress-train curves of NCF-NP nanofoam in *xy* and *z* direction, respectively. (**c**) Modulus of NCF monolith and NCF-NP1.0 nanofoam with different initial NCF. (**d**) Thermal conductivity of NCF and NCF-NP1.0 nanofoam in *z* direction.

The compressive stress-strain measurements in Fig. 3a,b display the typical deformation behavior of NCF-NP nanofoam with different NCF density (0.118, 0.163, 0.192, and 0.227 g/cm^3^). The initial sections of both curves increase linearly up to the point A which is taken as the compressive strength. The compressive strength increases from 1.337 to 2.940 MPa in *xy* direction and 0.577 to 1.592 MPa in *z* direction with densities of 0.257–0.346 g/cm^3^, respectively (Supplementary Table S4). The elastic deformation of this stage is believed to be formed through elastic bending and rotation of fibers. In the following non-elastic stage, the stress in xy direction decreasing with strain indicates that plastic instability occurs because of the buckling of bonded fibers. However, in z direction, linear elastic behavior at low strain, followed by a nanofoam collapse-related stress reduction at intermediate strains, and finally a plastic yielding plateau with subsequent stiffening at high strain^44^. The NCF-NP nanofoam is inclined to be tightly compressed and thus absorb more external energy, because most carbon fibers in NCF are perpendicular to the compressive force in z direction.

The NCF-NP nanofoam has a compressive modulus of E = 42.2~80.1 MPa along xy direction and 15.3~18.7 MPa in z direction for NCF-NP nanofoams (Fig. 3c), which is significantly higher than the values for NCF in this study (E = 3.5~40.5 MPa in xy direction and 3.7~12.6 MPa in z direction). This high modulus for NCF-NP nanofoam is related to the addition of NP and the fact that NP makes the NCF surface and voids of fiber-fiber stiffer by in-situ crosslinking (Fig. 1c). The co-continuous interlock structure for NCF-NP nanofoam is likely to be responsible for preserving the structural integrity and mechanical strength under loading conditions.

The thermal conductivity of the NCF-NP nanofoam is observed to increase from 0.188–0.289 Wm^−1^K^−1^ and 0.125–0.154 Wm^−1^K^−1^ in *xy* and *z* direction with an increase density of NCF from 0.118 to 0.227 g/cm^3^. The examined thermal conductivity is much lower than that of commercial carbon foam or traditional C-Ph composites, and actually comparable to that of glass fiber and carbon aerogels^45^,^46^. Noticeably, in z direction, chopped carbon fibers appear to be aligned and form a quasi-layered structure perpendicular to the direction of heat flow, which can enhance the thermal resistance. Moreover, Due to the formed fiber-aerogel-fiber pattern in NCF-NP nanofoam, the thermal transfer of fiber-fiber in NCF is separated by the NP aerogels, which helps to decrease heat-bridge and decrease the thermal conductivity. Though the use of nanosized NP may impart a significant interfacial thermal resistance, the increased density of NCF will decrease the porosity (Supplementary Table S5), and therefore the solid thermal conduction of NCF-NP will increase to a certain extent.

### Flame retardancy of NCF-NP nanofoam

Conventional polyurethane and polyethylene foam insulation materials are easily ignitable, and require the addition of flame retardants. Unfortunately, many of the commonly used flame retardants are halogenated or phosphorous compounds with negative environmental and health impacts^47^. Recent work has verified that the fire retardancy of NCF-NP nanofoams display very good fire retardancy, where the flame does not self-propagate. For NCF0.192-NP0.5, NCF0.192-NP1.0, NCF0.192-NP2.0 nanocomposite foams, the limiting oxygen index (LOI), which gives the oxygen concentration (in %) needed to keep a material burning, is as high as 34, 34 and 32, respectively, which is 60% higher than the O_2_ level in air (21%). The as-prepared NCF-NP nanofoams have a significantly higher LOI value than commercial, flame retardant-containing polymer-based foams.

**Figure 4:**
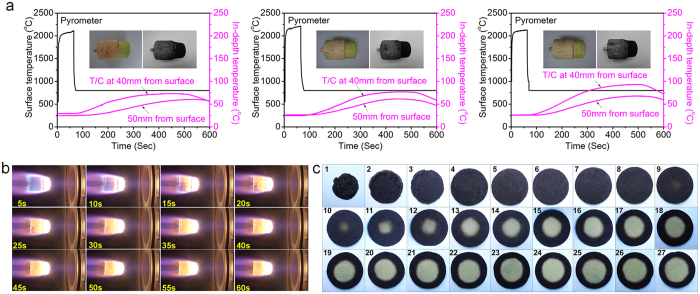
Wind tunnel testing of NCF-NP nanofoam. (**a)** Surface and internal temperature responses measured by a non-contact pyrometer and thermocouples located at 40 and 50 mm from initial stagnation point during the ablation and corresponding heat-sink period of NCF0.192-NP0.5, NCF0.192-NP1.0 and NCF0.192-NP2.0, respectively. Insert: Sample before and after ablation test. (**b**) Screenshot of sample NCF0.192-NP1.0 during ablation test recorded by camera at 5 to 60 sec. (**c**) Photograph of transverse slices of NCF0.192-NP1.0 after the ablation test. As evident from photographs, it is possible to visually estimate the transition from char to pyrolysis zone to virgin material for NCF-NP composites and observed material color approaching the virgin material at slice 9 in the near stagnation core.

To further evaluate the flame and fire-resistance of NCF-NP nanofoam under extreme environment, the flame-retardant behaviour was assessed with air-containing flame plasma arc tunnel. NCF-NP nanofoams were exposed to a defined heat flux of 3.7 MW/m^2^, and it was found the surface temperature quickly reached over 2000 °C after the three-group samples exposure. However, the internal temperature rise of NCF0.192-NP1.0 sample approximately reaches 77.3 °C and linear ablation rate is 0.057 mm/s, which is much lower than that of NCF0.192-NP2.0 sample (i.e., 93.3 °C and 0.063 mm/s) at 40 in-depth position from the surface during 500 seconds (Table S6). The resulted good heat blocking property and flame-retardancy is attributed to a higher T_d,max_ and maximum char yield for NP1.0 compared to NP0.5 and NP2.0, therefore the surface radiation will take away more heat and heat conduction can be inhibited. Figure 4b shows the flame ablation images of NCF0.192-NP1.0 nanocomposite during 60 s heating time, and the surface morphology of the tested aerodynamic shape is essentially unchanged besides the appearance of small recession after thermal ablation (see Supplementary Table S6). For conventional high and mid-density C-Ph composite, when flame heated, the generating gaseous products generally produce a reduction of convective heating. Chemical reactions can be endothermic (vaporization, sublimation) or exothermic (oxidation) and will lead to surface recession and charred structure. This result shows the NCF-NP nanofoam behaves a super thermal insulation performance and comparable ablation recession that assessed in wind tunnel or oxy-acetylene flame^48^,^49^,^50^,^51^,^52^.

Figure 4c exhibits photographs of transverse slices of NCF0.192-NP1.0 nanofoam after flame plasma test. As evident from Fig. 4c, it is possible to visually estimate the transition from char to pyrolysis to virgin matrix for NCF-NP nanofoam. Visual observations on transitions in nanofoam from segments agree with the density value generated for each slice. We have observed densities approaching the virgin matrix at position of slice 11, which further verifies the efficient thermal insulation capability of NCF-NP nanofoam.

**Figure 5:**
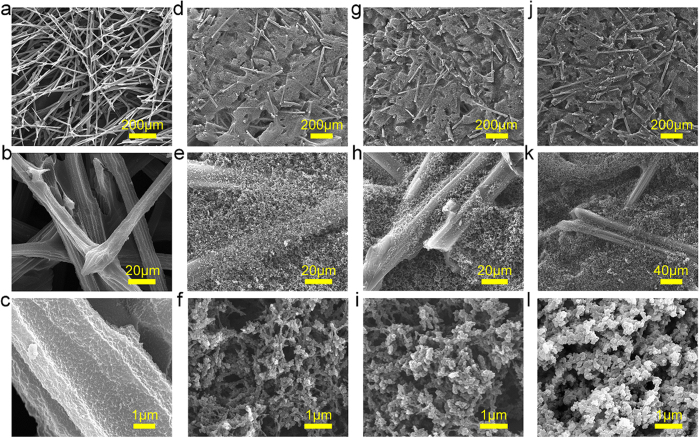
SEM of transverse slices of NCF0.192-NP1.0 at different height after the ablation test, (**a–l**) were taken in the centre part of slice, number 1, 3, 11, and 27 in Fig. 4c.

Figure 5 shows typical morphology as a function of depth of ablated NCF-NP nanofoam. Note that the morphology (Fig. 5a–c) of charred NCF-NP at the surface (about ~1.5 mm of the surface char) does not contain the NP phase at all other locations imaged, and the degradation of fibers present in the char can be observed, as the fiber diameter of the char is not comparable to that of the virgin fiber used to make NCF-NP nanofoam. The residual fibers still maintain 3D network bonded with each other, preventing them to blow away by the plasma flow and to inhibit the removal the fibers from the ablation surface^53^,^54^.

Figure 5(d–f) is the charred morphology composed of the fibrous NCF carbon perform and of the carbonized phenolic resin. A carbonaceous char layer was formed after thermal degradation of the NP, the formation of which has long been recognized as an efficacious way of enhancing the flame-retardancy of polymers. Char formation, being an endothermic process, critically defines the heat dissipation properties of ablative composite. A char layer dissipates a large fraction of the incident convective heat flux through surface radiation [61]. Therefore, along with efficient char formation, char retention for an extended duration guarantees better thermal insulating properties during thermal heating^55^,^56^,^57^,^58^,^59^.

Figure 5(g–i) is the pyrolyzed morphology of NCF-NP nanofoam. During pyrolysis, the phenolic resin is progressively carbonized into a low-density carbon or charred and loses around 50% of its mass, producing pyrolysis gases (i.e., CH_4_, CO, and H_2_). NCF-NP nanofoam remains its virgin state (Fig. 5(j–l)), which is made of a fibrous carbon perform consolidated by a micro- and meso-porous and high surface area NP.

## Discussion

A general strategy for fabrication of NCF-NP nanofoam with high-performance thermal insulation and super flame retardancy is presented with phenolic resin/HMTA/EG precursor solution by polymerization-induced phase separation. The co-continuous interlock structure for NCF-NP nanofoam includes nanoporous structured phenolic resin and 3D chopped carbon network, which is responsible for preserving the structural integrity and mechanical strength. The anisotropic thermal and mechanical properties of the prepared NCF-NP nanofoams are suitable for applications such as high-temperature fire-retardance in field of advanced buildings and aerospace thermal protection, where the high strain in the z direction and a low thermal conductivity in the z direction can be fully utilized. The as-prepared NCF-NP nanofoam has good thermal stability (T_d.max_ up to 536.27 °C). NCF-NP nanofoam exhibits low thermal conductivity (as low as 0.125 Wm^−1^K^−1^), and super flame-retardance under arc-jet wind tunnel simulation environment (small ablation recession, surface temperature up to 2200 °C and internal temperature below 60 °C at 40 mm in-depth position). These results provide substantial motivation to continue the development of high-performance lightweight thermal insulating and flame-retardant nanocomposites based on widely abundant resources for the improvement of energy efficiency and thermal protection/insulation.

## Methods

### NCF preparation

The fibrous low-density 3D chopped network-structured carbon fiber (NCF) monoliths have porosity up to 90% and were produced by carbonization (in argon atmosphere at 1000 °C for 1 h) of mixtures of chopped rayon based carbon fiber (1.6 mm in length) and powdered phenolic resin (200 mesh) attained by pressure filtration technique^19^. The NCF was washed with isopropyl glycol (IPA, 99.5%), followed by drying in air-circulated oven (50 °C for 1 h). To investigate the loading level of NCF affect the mechanical as well as flame retardant performance of NCF-NP nanofoam, NCF with densities of 0.118, 0.163, 0.192, and 0.227 g/cm^3^ were fabricated and named as NCF0.118, NCF0.163, NCF0.192, and NCF0.227, respectively.

### Fabrication of NCF-NP nanocomposite

Dried NCF monoliths (approximate diameter, 70 mm) were immersed in 300 ml of freshly prepared transparent resin solution of phenolic resin (PR), ethylene glycol (EG) and hexamethylenetetramine (HMTA) with weight ratio of EG/PR = 4. These were assisted by impregnating in vacuum chamber to ensure that the solution fully infiltrated in the carbon fabrics. The aforementioned four NCF and three different solutions with HMTA concentrations (ratio of HMTA to total weights of EG and PR) of 0.5% (sample NP0.5), 1.0% (sample NP1.0) and 2.0% (sample NP2.0) were used. The systems were heated at 90 °C for 1 h, and then 120 °C for 3 h, and lastly 180 °C for 3 h to bring about phase separation in the PR/HMTA/EG solution and further curing NP. The color of the resin samples changed from reddish brown to buff as nanoparticles formed in the co-precursor solution. The wet NP impregnated NCF artefacts were immersed in IPA to exchange the EG, and dried directly in air under ambient pressure at room temperature (≤30 °C) until constant weight was obtained. This resulted in NCF-NP composites with densities in the range of 0.247 to 0.346 g/cm^3^, and they were denoted as NCFx-NPy, where x is the density of NCF framework and y is the concentration of HMTA in the solution, respectively.

### Characterization

The surface morphology was obtained by field emission scanning electron microscopy (FESEM, Quanta 200 F). The elemental compositions and chemical binding states were analyzed by X-ray photoelectron spectroscopy (XPS, Escalab 250) and Fourier transform infrared spectroscopy (FTIR, PerkineElmer2000) using KBr disks. The thermal stability of aerogel was characterized by thermogravimetric analyses (TG/DSC, STA-409C, Netzsch) at a rate of 10 °C/min under Argon. N_2_ adsorption and desorption isotherms were taken using a surface area analyzer. The specific surface area was calculated by the Brunauer-Emmett-Teller (BET) method. Using the Barrett-Joyner-Halenda (BJH) model, the pore size distribution was derived from the desorption branch of the nitrogen adsorption isotherm. Before N_2_ adsorption, the samples were degassed at 100 °C for 24 h until the mass attained a constant value. Compression tests were performed on samples with dimensions of 10 × 10 × 12 mm^3^ using an Instron 5569 test stand at a compression rate of 0.5 mm/min. The thermal conductivity was determined by the Hot Disk TPS 2500 thermal constant analyzer at room temperature with sample with dimensions of 30 × 30 × 20 mm^3^.

The limiting oxygen index (LOI) was measured using a Dynisco LOI instrument, with the bar dimensions of 100 mm × 10 mm × 10 mm, according to the international standard ASTM D2863.The flame retardancy in z direction was evaluated in arc-jet facility at cold wall heat flux of 3.7 MW/m^2^, enthalpy of 35 MJ/kg and surface pressure of 2.5 kPa for 60 seconds with iso-Q sample. The sample was a cylinder with a spherical nose, the nose radius and the diameter were both 40.0 mm, and length was 50 mm. Surface and internal temperatures were measured by infrared pyrometer and K-type thermocouples. Also, the changes in shape during the testing process were also received and recorded by camera.

## Additional Information

**How to cite this article**: Cheng, H. *et al.* Super flame-retardant lightweight rime-like carbon-phenolic nanofoam. *Sci. Rep.* **6**, 33480; doi: 10.1038/srep33480 (2016).

## Supplementary Material

Supplementary Information

## Figures and Tables

**Table 1:** Textual characteristics of the prepared porous phenolic resin.

Sample	S_BET_ (m^2^/g)	S_mic_ (m^2^/g)	S_meso_ (m^2^/g)	V_meso_ (cm^3^/g)	D_average_ (nm)	T_5%_ (°C)	T_10%_ (°C)	T_d,max_ (°C)	R_1000 °C_ (%)
NP0.5	104.546	5.356	90.550	0.212	5.845	373.6	452.3	530.2	56.4
NP1.0	60.366	5.541	46.568	0.121	5.469	408.0	467.1	536.3	58.9
NP2.0	51.268	0.614	40.360	0.089	4.876	339.3	432.6	521.4	54.8

Note: S_BET_: BET surface area; S_mic_: Micropore surface area; S_meso_: BJH Adsorption cumulative surface area of pores between 1.7 and 300 nm diameter; V_meso_: BJH desorption cumulative pore volume of pores between 1.7 and 300 nm diameter; D_average_: Adsorption average pore width. T_5%_ and T_10%_: Thermal decomposition temperature at 5% and 10% weight loss; T_d,max_: Maximum rate of the weight loss; R_1000 °C_: Residue weight at 1000 °C.
