# Supplementary material for: Super flame-retardant lightweight rime-like carbon-phenolic nanofoam
Source: Sci Rep. 2016 Sep 15;6:33480. doi: 10.1038/srep33480 (PMC5024163; doi:10.1038/srep33480)
Supplement: Supplementary Information [file srep33480-s1.doc]

**Supplementary Material for**

**Super flame-retardant lightweight rime-like carbon-phenolic nanofoam**

**Haiming Cheng, Changqing Hong*, Xinghong Zhang*, Huafei Xue, Songhe Meng, Jiecai Han***

National Key Laboratory of Science and Technology on Advanced Composites in Special Environments, Harbin Institute of Technology, Harbin 150001, P.R. China

**Table S1** Experimental conditions of highly porous NP synthesis

| Sample | HMTA  (wt. % to PR+EG) | Phenolic resin  (wt. %) | EG  (wt. %) | Bulk density  (g/cm^3^) | Porosity  (%) |
| --- | --- | --- | --- | --- | --- |
| NP0.5 | 0.5 | 20 | 80 | 0.136 | 86.47 |
| NP1.0 | 1.0 | 20 | 80 | 0.139 | 86.27 |
| NP2.0 | 2.0 | 20 | 80 | 0.143 | 85.88 |

**Table S2** XPS analysis data of NP with different HMTA content

| Sample | Binding Energy Peak (eV) | Peak Area | Peak Area Ratio | Chemical bond |
| --- | --- | --- | --- | --- |
| NP0.5 | 399.612 | 1985.128 | 80.72% | HNC2 |
|  | 401.529 | 474.224 | 19.28% | NC3(4) |
| NP1.0 | 399.308 | 2742.365 | 92.97% | HNC2 |
|  | 401.793 | 207.509 | 7.03% | NC3(4) |
| NP2.0 | 399.184 | 4908.496 | 84.85% | HNC2 |
|  | 401.00 | 876.124 | 15.15% | NC3(4) |

**Table S3** The element composition of NP obtained from XPS spectrums.

| Sample | C | N | O |
| --- | --- | --- | --- |
| NP0.5 | 85.78 | 1.64 | 12.58 |
| NP1.0 | 84.41 | 2.17 | 13.42 |
| NP2.0 | 83.16 | 4.86 | 11.99 |

**Table S4 Mechanical properties of NCF-NP composites in xy and z directions.**

| Sample | xy direction | | z direction | | Ѱc |
| --- | --- | --- | --- | --- | --- |
|  | Strength  (MPa) | Modulus  (MPa) | Strength  (MPa) | Modulus  (MPa) |  |
| NCF0.118 | 0.376±0.078 | 3.454±0.216 | 0.140±0.023 | 3.683±0.4707 | 2.685 |
| NCF0.163 | 0.719±0.204 | 22.963±4.533 | 0.323±0.087 | 6.986±0.563 | 2.226 |
| NCF0.192 | 0.855±0.091 | 32.117±6.052 | 0.405±0.028 | 8.919±0.529 | 2.109 |
| NCF0.227 | 1.704±0.246 | 40.496±6.838 | 0.776±0.085 | 12.748±4.488 | 2.195 |
| NCF0.118-NP1.0 | 1.337±0.113 | 42.212±8.307 | 0.577±0.043 | 18.733±2.566 | 2.317 |
| NCF0.163-NP1.0 | 1.637±0.252 | 48.381±9.019 | 0.759±0.054 | 15.329±0.560 | 2.157 |
| NCF0.192-NP1.0 | 2.053±0.116 | 58.867±14.503 | 0.953±0.052 | 17.200±1.648 | 2.154 |
| NCF0.227-NP1.0 | 2.940±0.505 | 80.147±7.584 | 1.592±0.052 | 17.214±2.368 | 1.847 |

**Table S5** Physical properties of NCF-NP composites preparation

| Sample | Bulk density of NCF  (g/cm^3^) | Porosity of NCF  (%) | Bulk density of NCF-NP  (g/cm^3^) | Porosity  (%) | Content of NCF  (vol. %) | Content of NP  (vol. %) |
| --- | --- | --- | --- | --- | --- | --- |
| NCF0.118-NP1.0 | 0.118 | 92.6 | 0.247 | 81.9 | 7.4 | 10.7 |
| NCF0.163-NP1.0 | 0.163 | 89.8 | 0.288 | 79.4 | 10.2 | 10.4 |
| NCF0.192-NP0.5 | 0.192 | 88.0 | 0.312 | 78.0 | 12.0 | 10.0 |
| NCF0.192-NP1.0 | 0.192 | 88.0 | 0.314 | 77.8 | 12.0 | 10.2 |
| NCF0.192-NP2.0 | 0.192 | 88.0 | 0.319 | 77.4 | 12.0 | 10.6 |
| NCF0.227-NP1.0 | 0.227 | 85.8 | 0.346 | 75.9 | 14.2 | 9.9 |

**Table S6 Wind tunnel testing results of NCF-NP nanocomposites**

| Sample | Lo/La  (mm) | R_L_  (mm/s) | Mo/Ma  (g) | R_M_  (g/s) | Temperature peak (^o^C) | | |
| --- | --- | --- | --- | --- | --- | --- | --- |
|  |  |  |  |  | Surface | T/C at 40mm | T/C at 50mm |
| NCF0.192-NP0.5 | 50.00/46.45 | 0.059 | 20.43/12.17 | 0.1376 | 2132.3 | 73.2 | 60.4 |
| NCF0.192-NP1.0 | 50.00/46.58 | 0.057 | 20.57/12.38 | 0.1365 | 2215.4 | 77.3 | 61.5 |
| NCF0.192-NP2.0 | 50.00/46.22 | 0.063 | 20.78/10.83 | 0.1658 | 2114.6 | 93.3 | 70.0 |

Note: Lo and La is thickness of specimen measured before and after ablation test; Mo and Ma is mass of specimen before and after ablation test; R_L_ and R_M_ is recession rate and mass loss rate, was calculated by thickness and mass change before and after ablation test of each specimen using R_L_=(Lo-La)/t and R_M_=(Mo-Ma)/t, where t is ablation time (s).

**
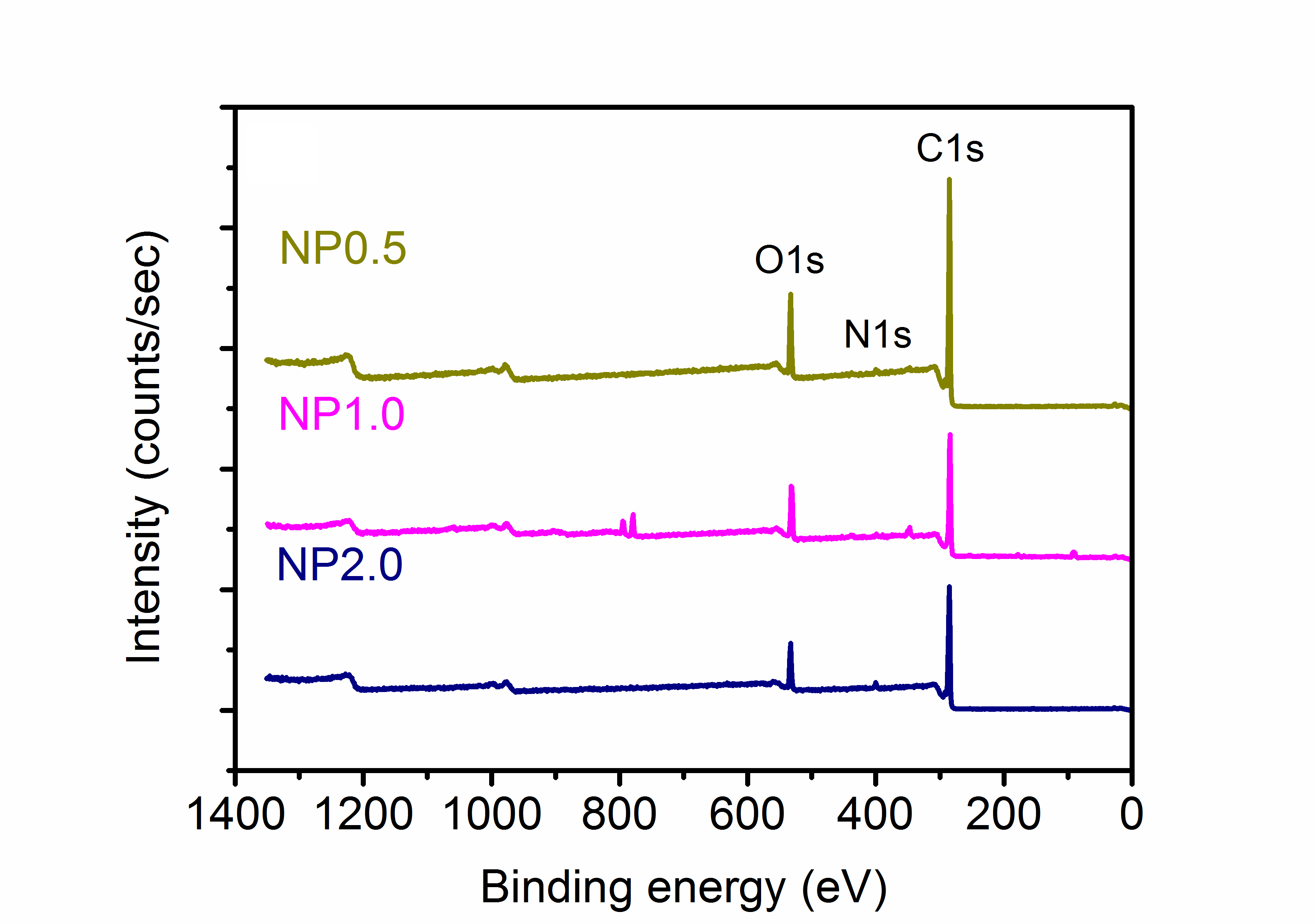
**

**Fig. S1** Full-scan XPS spectra of sample NP0.5, NP1.0 and NP2.0.


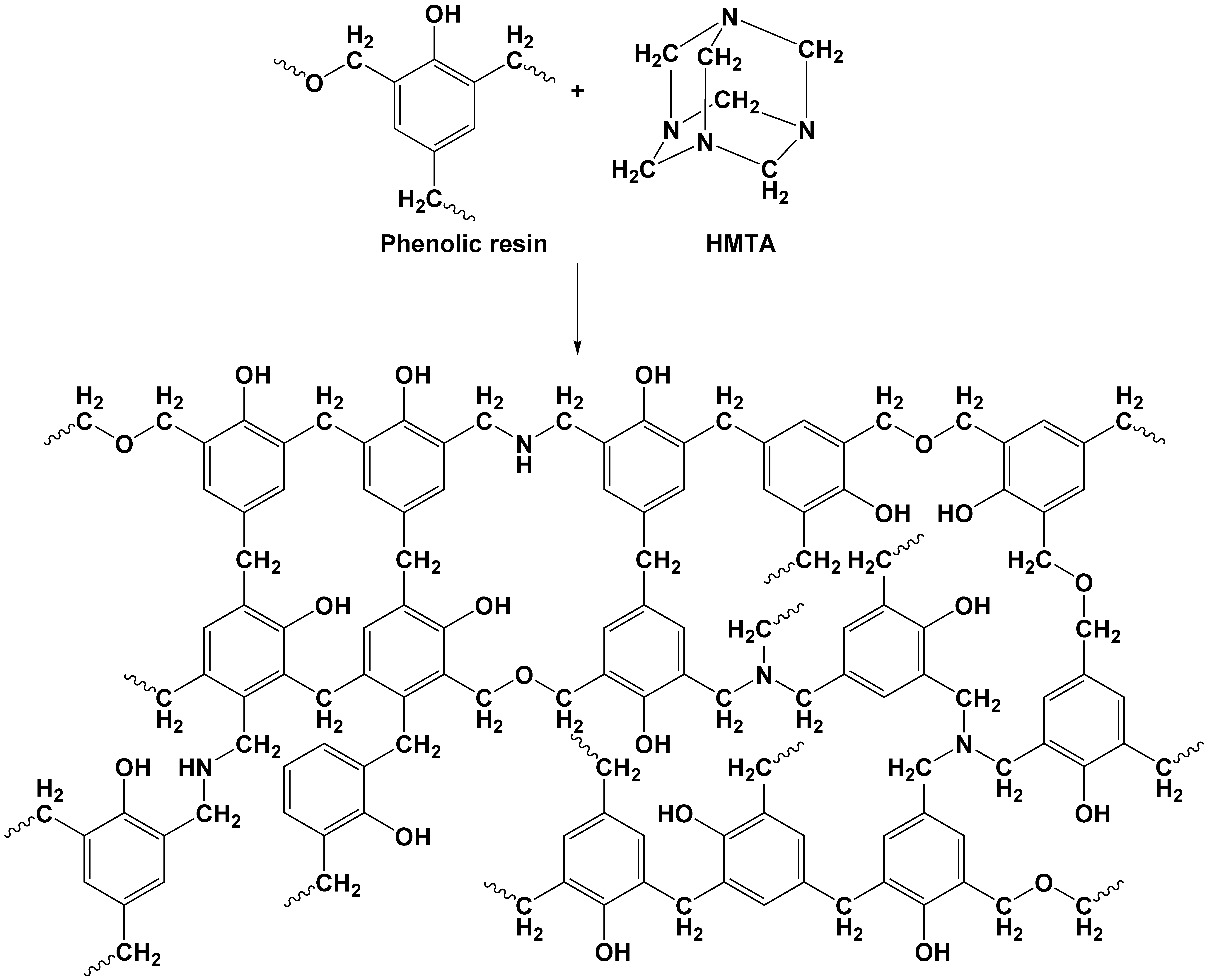


**Fig. S2** The proposed molecular structure of NP obtained from a phnolic solution containing phenolic resin, HMTA and EG as coprecursors.
